# Integrative Activity of Mating Loci, Environmentally Responsive Genes, and Secondary Metabolism Pathways during Sexual Development of Chaetomium globosum

**DOI:** 10.1128/mBio.02119-19

**Published:** 2019-12-10

**Authors:** Zheng Wang, Francesc López-Giráldez, Junrui Wang, Frances Trail, Jeffrey P. Townsend

**Affiliations:** a Department of Biostatistics, Yale School of Public Health, New Haven, Connecticut, USA; b Yale Center for Genome Analysis, Yale University, New Haven, Connecticut, USA; c Department of Genetics, Yale University, New Haven, Connecticut, USA; d Department of Plant, Soil and Microbial Sciences, Michigan State University, East Lansing, Michigan, USA; e Department of Ecology and Evolutionary Biology, Yale University, New Haven, Connecticut, USA; f Program in Computational Biology and Bioinformatics, Yale University, New Haven, Connecticut, USA; University of Melbourne

**Keywords:** Bayesian network, environmental response, fungal pathogen, homothallism, secondary metabolism, sexual development, transcriptomics

## Abstract

The origins and maintenance of the rich fungal diversity have been longstanding issues in evolutionary biology. To investigate how differences in expression regulation contribute to divergences in development and ecology among closely related species, transcriptomes were compared between Chaetomium globosum, a homothallic pathogenic fungus thriving in highly humid ecologies, and Neurospora crassa, a heterothallic postfire saprotroph. Gene expression was quantified in perithecia at nine distinct morphological stages during nearly synchronous sexual development. Unlike N. crassa, expression of all mating loci in C. globosum was highly correlated. Key regulators of the initiation of sexual development in response to light stimuli—including orthologs of N. crassa *sub-1*, *sub-1*-dependent gene NCU00309, and *asl-1*—showed regulatory dynamics matching between C. globosum and N. crassa. Among 24 secondary metabolism gene clusters in C. globosum, 11—including the cochliodones biosynthesis cluster—exhibited highly coordinated expression across perithecial development. C. globosum exhibited coordinately upregulated expression of histidine kinases in hyperosmotic response pathways—consistent with gene expression responses to high humidity we identified in fellow pathogen Fusarium graminearum. Bayesian networks indicated that gene interactions during sexual development have diverged in concert with the capacities both to reproduce asexually and to live a self-compatible versus self-incompatible life cycle, shifting the hierarchical roles of genes associated with conidiation and heterokaryon incompatibility in N. crassa and C. globosum. This divergence supports an evolutionary history of loss of conidiation due to unfavorable combinations of heterokaryon incompatibility in homothallic species.

## Abstract (precis)

Fungal diversity has amazed evolutionary biologists for decades. One societally important aspect of this diversity manifests in traits that enable pathogenicity. The opportunistic pathogen Chaetomium globosum is well adapted to a high-humidity environment and produces numerous secondary metabolites that defend it from predation. Many of these chemicals can threaten human health. Understanding the phases of the C. globosum life cycle in which these products are made enables better control and even utilization of this fungus. Among its intriguing traits is that it both is self-fertile and lacks any means of propagule-based asexual reproduction. By profiling genome-wide gene expression across the process of sexual reproduction in C. globosum and comparing it to genome-wide gene expression in the model filamentous fungus N. crassa and other closely related fungi, we revealed associations among mating-type genes, sexual developmental genes, sexual incompatibility regulators, environmentally responsive genes, and secondary metabolic pathways.

## INTRODUCTION

Fungi encompass an enormous morphological diversity that is distributed across almost every environment on earth (1). Understanding the evolution of such diversity has become one of the central issues in evolutionary developmental biology (2–4). Fungi of the Sordariomycetes include the genetic, pathogenic, and developmental models Neurospora crassa, Fusarium graminearum, and Sordaria macrospora. These model fungi have enabled the identification of numerous genes critical for sexual development via comparative genomics and transcriptomics (5–10). Among the Sordariomycetes are numerous examples of heterothallic (self-incompatible) and homothallic (self-compatible) species. Nevertheless, we have yet to understand how heterothallism and homothallism affect the whole sexual developmental process.

The genus *Chaetomium* (Chaetomiaceae, Sordariales) includes about 100 species identified from diverse high-humidity environments, including aquatic niches (11–13). *Chaetomium* perithecia have a few features that have been suggested to be adaptive to their highly humid habitats, including a characteristic membranaceous wall covered by conspicuously flexuous long hairs (14). Single-celled, smooth, pigmented ascospores are released from asci inside the perithecium, then squeezed out through the ostiole, and trapped by the coiled hairs (15). Chaetomium globosum, the type species of the genus, occupies diverse substrates (aerial, terrestrial, aquatic, marine, and pathogenic [16, 17]). Whereas most pathogenic fungi rely on asexual reproduction of conidia as a major distribution strategy, asexual spores have not been observed in C. globosum. Perithecial hairs of *Chaetomium* species have been attributed defensive value as physical barriers against predatory insects (18) and in functioning as mucilage-filled sacs that enable flotation-based water transport during the maturation of the perithecia (19, 20).

C. globosum is a pathogen of diverse animal and human hosts (21–29) and causes nail infections that have been increasing worldwide over recent decades (26, 28, 30). It produces a diverse array of secondary metabolites, including toxic chaetoglobosins, chaetomugilins, chaetoviridins, and cochliodones (31–34). These chemicals also have potential for applications in the biocontrol of pests and the treatment of cancer because of their high cytotoxicity (25, 35–40). A draft genome sequence of the self-compatible conditional human pathogen Chaetomium globosum—a close relative of N. crassa—was recently published, enabling comparative genomic investigation (41).

Research on mating activity in fungi has primarily focused on mating-type genes and their evolution (42–48). C. globosum is self-compatible, a derived feature exhibited by diverse fungal lineages (49, 50). The closely related genus *Neurospora* includes the pseudohomothallic model N. tetrasperma and two heterothallic models, N. crassa and N. discreta, for which two mating types are required for successful crossing and subsequent sexual development (51–55). Gene expression studies with these individual species have illuminated gene expression dynamics associated with pigmentation and meiotic sporulation (5, 6), perithecial development (5, 7), and responses to genetic and environmental signals (6). Several comparative studies have shed light on the evolution of gene expression and on the genetic basis of perithecium production (3, 5–7, 56, 57). Studies of several filamentous fungi (58–62) have investigated the genome-wide impacts of activities of mating-type and other sex-associated loci during sexual development. Early studies of genome-wide gene expression in Sordaria macrospora have indicated that mating-type loci might play a role in late sexual development (48, 63).

To investigate how differences in regulation of expression might contribute to divergence in development and ecology among closely related species, we conducted comparative transcriptomic sequencing of Chaetomium globosum, *Neurospora* spp. (5, 6, 64), and *Fusarium* spp. (7, 56). We compared expression of mating-type loci between C. globosum and N. crassa from both mating types across the entirety of perithecial development. We identified orthologous genes responsive to environmental signals among C. globosum, N. crassa, and F. graminearum and assessed their ancestrally retained, convergent, or divergent expression patterns and their unique habitat adaptations. We also analyzed the expression of secondary metabolism pathways. Unannotated genes that exhibited similar expression patterns between C. globosum and N. crassa with peak expression during ascus and ascospore development were selected, and their knockout phenotypes were characterized in N. crassa.

## RESULTS

A total of 5,784 single-copy orthologs were identified by comparison of C. globosum CBS 148.51 and N. crassa *mat A* OR74A genomes (see Table S1 in the supplemental material). The ortholog of mating-type gene *mta-1* (encoding MAT1-2-1) in C. globosum was identified as the *mat a* gene in the heterothallic N. crassa strain trp-3 and in pseudohomothallic *N. tetrasperma* strain P0656.

10.1128/mBio.02119-19.5 TABLE S1 Single-copy orthologs identified between Chaetomium globosum and Neurospora crassa genomes and, where available, KEGG functional annotation. Download Table S1, XLSX file, 0.4 MB. Copyright © 2019 Wang et al. 2019 Wang et al. This content is distributed under the terms of the Creative Commons Attribution 4.0 International license.

### Morphology and development.

**FIG 1:**
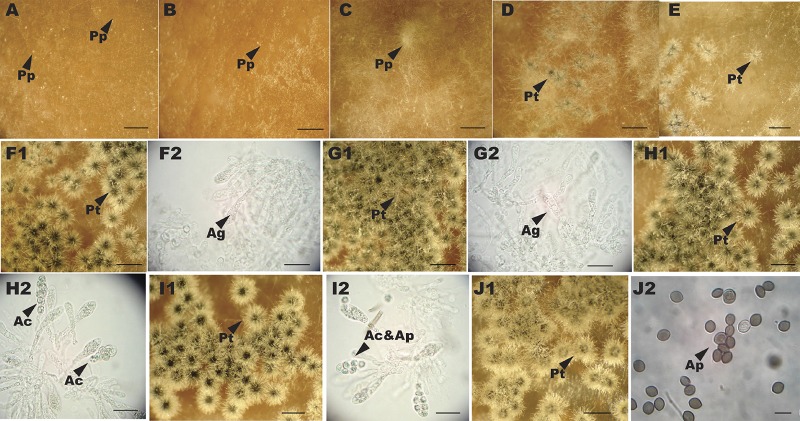
Stages of sexual development of Chaetomium globosum, viewed with a Leica S60 stereomicroscope and Leica CM E microscope. (A to E) Protoperithecia imaged with SONY DSC-H2 camera prior (0 h) (A) and subsequent to (B) disturbance and perithecial development at stages 2 to 4 (2 h, 24 h, and 48 h) (C to E). (F to J) Morphology of perithecial development at stages 5 to 9 (72, 96, 120, 144, and 168 h). Shown are macroscopic images of cultures (F1 to J1) and light microscope images (F2 to I2; scale bars: 50 μm) of perithecial squashes mounted in water. Arrows identify protoperithecia (Pp), perithecia (Pt), ascogenous tissues (Ag), asci (Ac), and ascospores (Ap). Scale bars: 1 mm.

Five days after hyphal fragments filtered from liquid culture were placed on carrot medium, C. globosum developed abundant pale protoperithecia with a few hairs (Fig. 1 and Fig. S1). The first stage (at 0 h) was defined as having the majority (>50%) of protoperithecia well developed. Physical disturbance of a light swipe of the plate surface covered with protoperithecia, as described in references 6 and 56, imposed a synchronization of perithecial development at the second stage (2 h). At stages 3 to 9 (24, 48, 72, 96, 120, 144, and 168 h), the majority of sexual reproductive structures had reached respective developmental stages characterized by young perithecia (abundant hairs or dark colored), double-sized perithecia with thin-walled ascogenous cells, asci containing condensed spore content, asci with light-colored young ascospores, mature asci with dark ascospores, and released ascospores. Nearly 70% of perithecia matured and released ascospores within 8 days after appearance of protoperithecia (Fig. 1 and Fig. S1). Perithecial development of C. globosum and that of species of *Neurospora* are highly similar in terms of timing in appearance of major morphological characters (6, 7). The transcriptome was assayed at the additional stage of ascospore release (stage 9) in C. globosum, at which time sampled tissue on the cellophane membrane included both perithecia and released ascospores. Spore release in N. crassa is a quick process, highly responsive to various environmental factors and challenging to precisely control; thus, the spore release stage was not sampled for N. crassa.

10.1128/mBio.02119-19.1 FIG S1 (A) Synchronization of perithecial development in Chaetomium globosum was aided via inoculation of solid carrot agar cultures by hyphal fragments from filtered liquid cultures. (B to D) Synchronized development of C. globosum perithecia on CA plates sampled at 5 (B), 8 (C), and 11 (D) days after inoculation. Download FIG S1, TIF file, 1.9 MB. Copyright © 2019 Wang et al. 2019 Wang et al. This content is distributed under the terms of the Creative Commons Attribution 4.0 International license.

### Gene expression across perithecial development.

**FIG 2:**
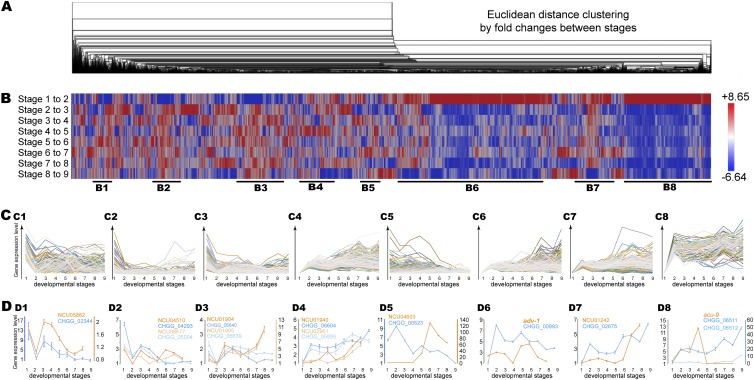
Genome-wide gene expression of Chaetomium globosum across sexual development, clustered with Euclidean distance and representative expression patterns. (A) Hierarchical gene clustering and corresponding expression patterns over sexual development, for which expression was compared as fold change. (B) Representative clusters showing predominant expression of subpatterns 1 to 8 (B1 to B8). (C) Gene expression profiles (relative to lowest stage-specific expression) for genes in each of subpatterns 1 to 8 (C1 to C8). (D) Comparison of expression between C. globosum (blue) and Neurospora crassa (orange) for selected orthologous gene pairs in each of subpatterns 1 to 8 (D1 to D8), suggesting ancestrally retained or recently divergent expression between the two fungi during sexual reproduction.

LOX (Level Of eXpression) yielded well-measured relative expression levels across more than one sampled stage for 11,170 of the predicted 11,232 genes in the C. globosum genome (Tables S2 and S3). Genome-wide gene expression of C. globosum across sexual development generally followed two frequent patterns (Fig. 2). One pattern started with downregulation, followed by upregulation toward the end of perithecial development (Fig. 2B1 to B3 and B5). The other pattern started with upregulation for the 2 h after disturbance, followed by downregulation toward the last stage of spore release (Fig. 2B4 and B6 to B8). Within these two general patterns, expression of genes can be further clustered into subpatterns 1 to 8 (Fig. 2B1 to B8 and Table S3). Few single-copy orthologs were detected that belong to the subpattern 1 (Fig. 2B1 and Fig. 2C1). For example, gene CHGG_02344 exhibited gene expression dynamics nearly identical to those of its N. crassa ortholog NCU05882 (Fig. 2D1).

10.1128/mBio.02119-19.6 TABLE S2 Sequencing and mapping of quality statistics for all transcriptome sequencing (RNA-seq) samples. Adapter sequences, empty reads, and low-quality sequences were removed. For each read, we trimmed the first 6 nucleotides, and trimmed the last nucleotides at the point where the Phred score of an examined base fell below 20 using in-house scripts. If, after trimming, the read was shorter than 45 bp, the entire read was discarded. Trimmed reads were aligned to the Chaetomium globosum genome from the NCBI database with its genome annotation using Tophat v.2.1.1, applying the very-sensitive preset and providing the corresponding gene model annotation. Only the reads that mapped to a single unique location within the genome, with a maximum of two mismatches in the anchor region of the spliced alignment, were reported. We used the default settings for all other Tophat options. We tallied reads by aligning to exons of genes with the program HTSeq v0.6.1p1. A tally of the number of the reads that overlapped the exons of a gene was calculated using aligned reads and the gene structure annotation file for the reference genome. In addition to the 17 libraries representing biological replicates, five technical replicates were sequenced to ensure quality control among serial sequencing runs. Download Table S2, XLSX file, 0.01 MB. Copyright © 2019 Wang et al. 2019 Wang et al. This content is distributed under the terms of the Creative Commons Attribution 4.0 International license.

10.1128/mBio.02119-19.7 TABLE S3 Genome-wide gene expression levels across sexual development in Chaetomium globosum. Download Table S3, TXT file, 2.4 MB. Copyright © 2019 Wang et al. 2019 Wang et al. This content is distributed under the terms of the Creative Commons Attribution 4.0 International license.

In general, genes that are critical for development showed similar degrees of regulation of expression between C. globosum and N. crassa. Examples included two late light-responsive genes in subpattern 2 (Fig. 2D2), a checkpoint kinase and a 3′-to-5′ exonuclease in replication and recombination in subpattern 4 (Fig. 2D4), as well as basal hyphal growth and asexual/sexual development regulator *adv-1* in subpattern 6 (Fig. 2D6). Interestingly, numerous genes with direct roles in metabolism exhibited divergent expression between C. globosum and N. crassa. Examples included secondary metabolism-related reductase and dehydrogenase genes in subpattern 3 (Fig. 2D3) and chitinase genes in subpattern 5 (Fig. 2D5), as well as a kinase-activating protein-coding gene in subpattern 7 (Fig. 2D7). Two C. globosum genes homologous to N. crassa *acu-9* exhibited upregulation at the end of perithecial development, whereas N. crassa *acu-9* exhibits downregulation starting at 48 h after crossing (Fig. 2D8).

### Regulation of melanin production.

**FIG 3:**
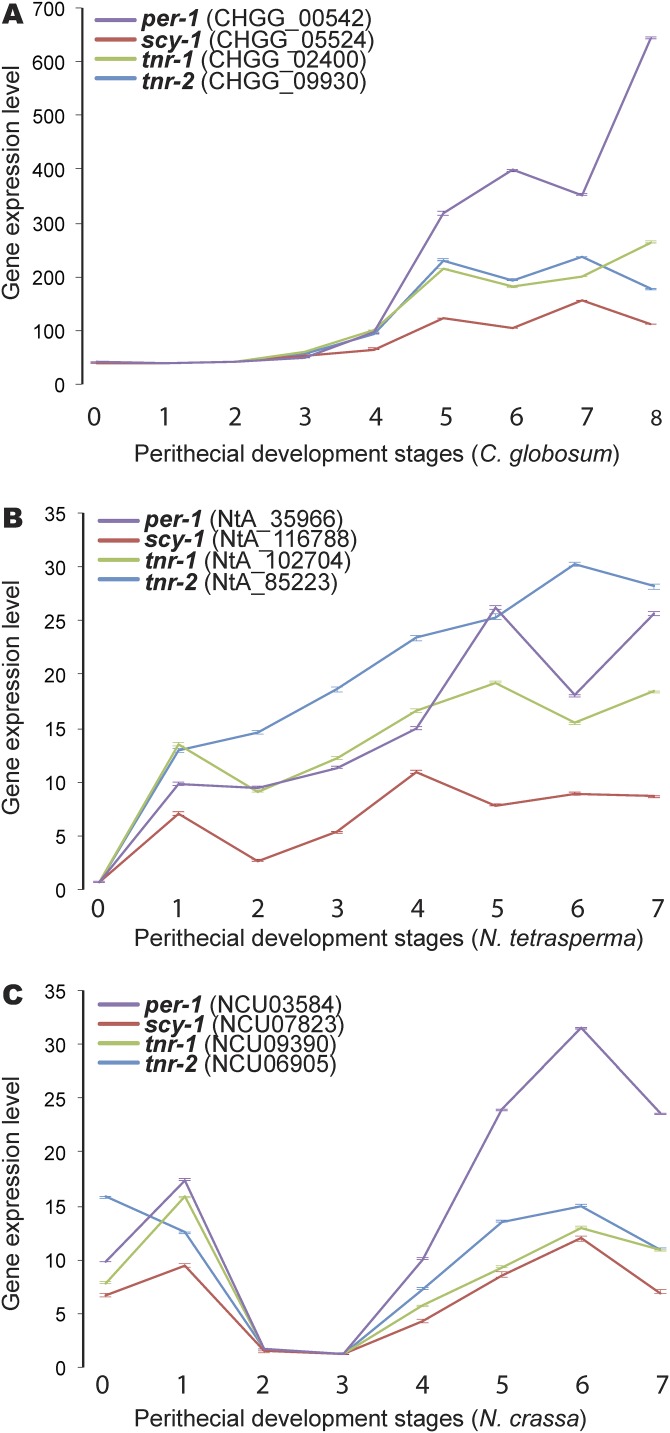
Gene expression of melanin synthases (fold change relative to the stage of lowest expression) exhibited upregulation as ascospores matured in Chaetomium globosum (A), Neurospora tetrasperma (B), and Neurospora crassa (C). Genes are identified by the name of the ortholog in N. crassa (*per-1*, *scy-1*, *tnr-1*, and *tnr-2*). Bars indicate 95% confidence intervals.

Dark pigmentation caused by melanin biosynthesis is a phenotypic marker of sexual development in many Sordariomycetes. Melanin biosynthesis is associated with four major enzymes: a polyketide synthase (*per-1*), scytalone dehydratase (*scy-1*), and tetrahydroxynaphthalene reductases 1 and 2 (*tnr-1* and *tnr-2*). Expression of these melanin biosynthesis genes across perithecial development was found to be highly coordinately regulated within each of the species (previously reported for N. crassa [6]). However, in contrast to the two-phase upregulation in *Neurospora* species (at the 2-h sampling point and later during ascospore development), expression of these genes exhibited a monotonic upregulation in C. globosum (Fig. 3).

### Expression of mating loci and pheromone genes.

**FIG 4:**
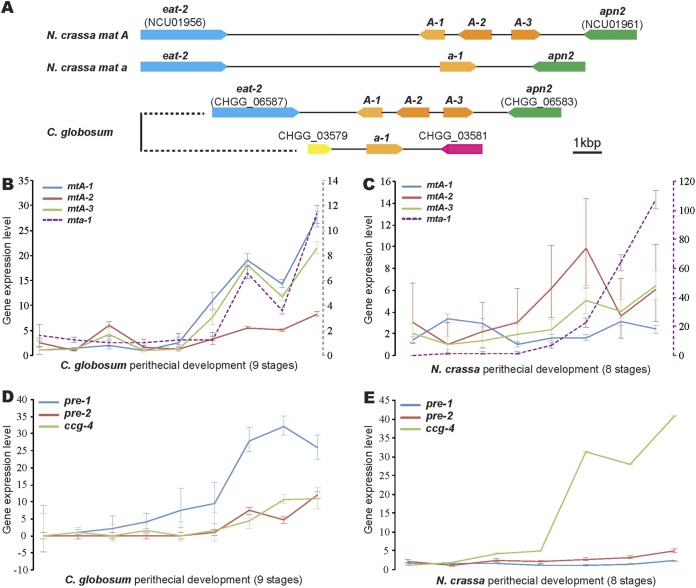
Comparison of mating loci and their expression in Chaetomium globosum and Neurospora crassa. (A) Mating-type regions annotated for C. globosum using N. crassa as a reference, showing conserved gene arrangements for *mat A* loci. The genes *eat-2* and *apn2* are not mating-type genes and are present in both mating types in N. crassa in a conserved order. C. globosum *mat a-1* is unlinked to *mat A* loci and bordered by genes for two hypothetical proteins: CHGG_03579, which is a partial duplicate of gene CHGG_06785 and similar (39.42%) to GE21Draft_1331765 in the N. crassa *mat a* genome, and CHGG_03581, similar (60.6%) to the *mat A-2* gene in C. globosum. (B to E) Expression of mating type **A** genes (*mtA-1*, *mtA-2*, and *mtA-3*), the mating type **a** gene (*mta-1*), and pheromone-related genes (*pre-1*, *pre-2*, and *ccg-4*) across sexual development. Note the second *y* axis (purple dashed line) provided to quantify relative expression of *mta-1* occurring on a different overall scale. Expression has been quantified relative to the lowest stage-specific expression level. Bars indicate 95% confidence intervals.

Orthologs of genes coding for all four mating-type proteins in N. crassa, including Mat A-1, Mat A-2, and Mat A-3 for the **A** strain and Mat a-1 for the **a** strain, coexist in the genome of C. globosum (Fig. 4A). Orthologs of N. crassa genes that code for the **A**-specific pheromone precursor CCG-4, and for two the pheromone receptors PRE-1 (responsive to CCG-4) and PRE-2 (responsive to MFA-1), exist in the C. globosum genome. N. crassa **a**-specific pheromone precursor *mfa-1* encodes a very short protein; no ortholog of *mfa-1* or a similar precursor protein was identified or annotated in C. globosum. In C. globosum, all four mating-type loci were highly coordinately regulated, especially during the late stages of sexual development (>48 h [Fig. 4B]). Expression among the three mating type **A** genes *mtA-1*, *mtA-2*, and *mtA-3* and that between them and the mating type **a** gene *mta-1* were inconsistent during sexual development in N. crassa (Fig. 4C). Expression of *mat a-1* was not expected and indeed was not detected in **A** protoperithecia in N. crassa and was almost undetectable before stage 4, but after stage 4 its expression increased monotonically, peaking at the last sampling stage of ascospore maturation in N. crassa at 107-fold above that at 0 h. In C. globosum, expression of *ccg-4* and *pre-1* exhibited similar degrees of upregulation toward spore maturation and release (Fig. 4D). In N. crassa, *ccg-4* expression exhibited a dramatic monotonic increase culminating in 45-fold upregulation over 0-h expression by stage 8 of perithecial development (Fig. 4E). However, expression levels of *ccg-4* do not necessarily reflect the pheromone level within the fungal culture: the pheromone genes it upregulates encode prepropheromones that require further posttranscriptional processing to yield mature pheromones.

### Regulators of sexual development in response to environmental signals.

**FIG 5:**
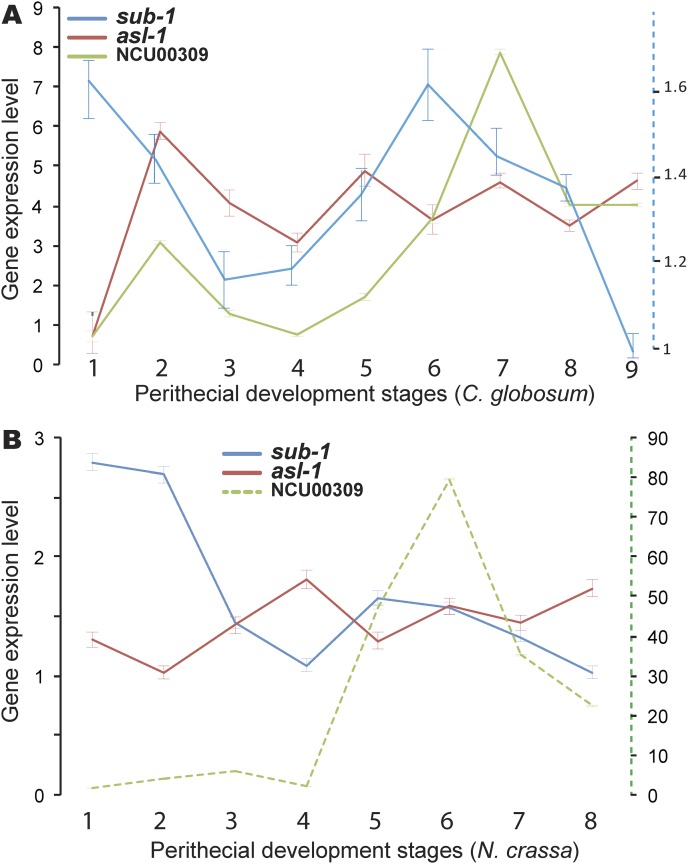
Three Chaetomium globosum genes and their orthologs in Neurospora crassa that are light responsive and critical to the initiation of N. crassa sexual reproduction exhibit similar expression dynamics in C. globosum (A) and N. crassa (B) during perithecial development. Bars indicate 95% confidence intervals. Note the second *y* axis (blue dashed line) provided to quantify relative expression of C. globosum ortholog of *sub-1* occurring on a different overall scale.

As has been demonstrated in several model fungal species, light serves as a key environmental signal of fungal development (8, 65, 66). Three light-responsive genes—submerged protoperithecia (*sub-1*), *sub-1*-responsive gene NCU00309 (67), and ascospore lethal (*asl-1*)—are required for normal sexual development in N. crassa. Expression of *sub-1* and NCU00309 orthologs in C. globosum exhibited a regulation pattern similar to that in N. crassa, only different in scale (Fig. 5). Gene *asl-1* is characterized by an undulating expression across sexual development in N. crassa (68, 69). Its ortholog in C. globosum exhibited a similar dynamic but appears to have phase shifted 24 h earlier: it peaked in expression at the second stage, whereas in N. crassa, *asl-1* expression dropped to its lowest point at the 2-h crossing time point.

Aside from developmental guidance obtained from environmental light signals (8, 65, 66, 70), sexual reproduction in many fungal species is regulated by numerous other environmental stress factors (70). Catalase genes are found in aerobically respiring organisms and function to protect cells from the toxic effects of hydrogen peroxide; three N. crassa catalase genes—*cat-1*, *cat-2*, and *cat-3*—are known to be highly expressed during asexual reproduction (71–75). Orthologs of these genes exhibited downregulated expression during perithecial development in C. globosum, in contrast to the coordinate upregulation (especially as perithecia and ascospores matured) observed in N. crassa (Fig. S2), for which airflow is a critical component of the successful dispersal of forcibly released ascospores.

10.1128/mBio.02119-19.2 FIG S2 Expression of catalase genes *cat-1*, *cat-2*, and *cat-3* exhibited divergent regulation between Chaetomium globosum (A) and Neurospora crassa (B), suggesting that perithecial development in these species has adapted to differing microenvironmental conditions such as oxygen level and airflow intensity. Note the second *y* axis (blue dashed line) provided to quantify relative expression of *cat-1* occurring on a different overall scale. Download FIG S2, TIF file, 1.0 MB. Copyright © 2019 Wang et al. 2019 Wang et al. This content is distributed under the terms of the Creative Commons Attribution 4.0 International license.

### Expression regulation for genes critical for asexual reproduction and heterokaryon incompatibility.

**FIG 6:**
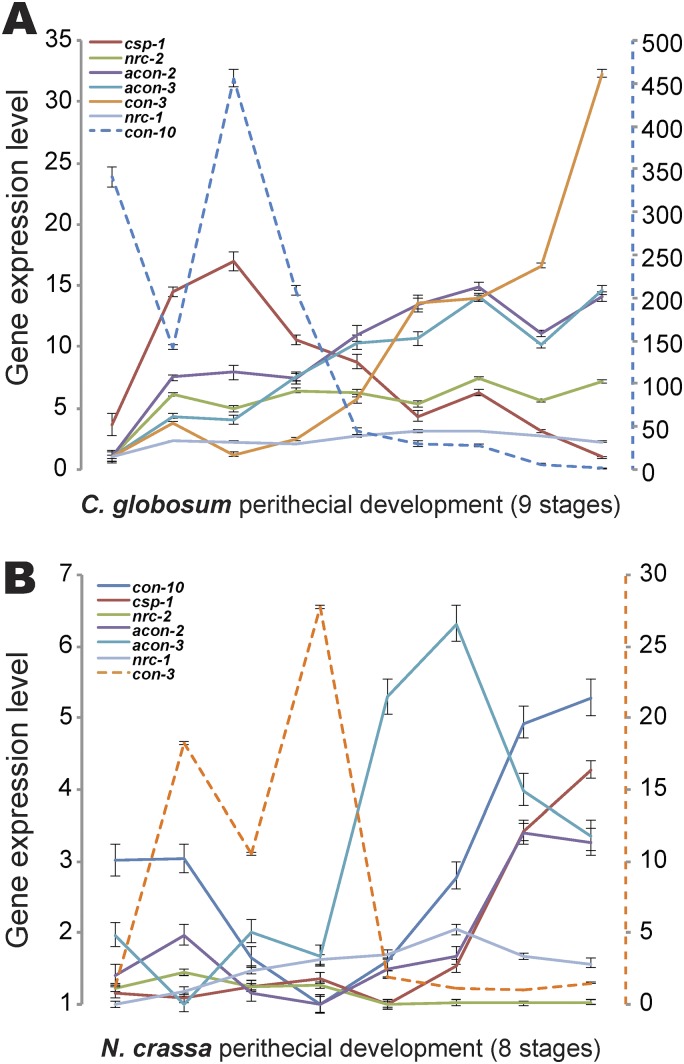
Divergent expression of genes critical for conidiation during sexual development, comparing orthologs in Chaetomium globosum (A) to genes of Neurospora crassa (B). Note the second *y* axis for *con-10* (blue dashed line) in C. globosum and *con-3* (orange dashed line) in N. crassa, provided to quantify relative expression occurring on a different overall scale. Bars indicate 95% confidence intervals.

Orthologs of some N. crassa conidiation genes were identified in the C. globosum genome, including *aconidiate-2* and *-3* (*acon-2* and *acon-3*), *conidiation-3* and *-10* (*con-3* and *con-10*), *non-repressor of conidiation-1* and *-2* (*nrc-1*, *nrc-2*), and *conidia separation-1* (*csp-1*). In N. crassa, genes *acon-2* and *acon-3* are required for macroconidiation (76), and their expression was upregulated during ascospore formation (stages 4 to 6); in C. globosum, expression of their homologs was upregulated across sexual development. Conidiation-associated *con-10* exhibits a dramatic expression response to light in conidiating tissues (77), and proliferative expression of *con-10* has also been reported during perithecial development of N. crassa (6, 66, 78). *csp-1*, a global circadian repressor, regulates membrane formation via ergosterol synthesis (79), while *nrc-2* is required to repress conidiation (80). In N. crassa, *csp-1* expression was upregulated during sexual development and *nrc-2* expression was consistently high. Expression of their orthologs in C. globosum contrasted: *con-10* and *csp-1* were both downregulated (Fig. 6). No orthologs of N. crassa *con-6*, *con-8*, *con-13*, or *fluffy* (*fl*) were identified in the C. globosum genome.

Multiple heterokaryon incompatibility (*het*) genes are fairly common in ascomycete genomes (81). Evolutionary analyses of the well-studied *het* genes in N. crassa—including two copies of *tol*, two copies of *het-C*, and *het-6*, *-13*, *-14*, and *-15*—distinguish three gene families, HET-6/13/15, HET-C, and HET14-TOL, composed of 22, 4, and 21 genes with clear homology between N. crassa and C. globosum (Fig. S3). No homologs of N. crassa Pin-C (NCU03494), an interacting protein with Het-C, were identified in C. globosum. Along with more than half of the genome being upregulated (4,983/9,717 genes), expression of 15 (out of 24; *P = *0.31, Fisher exact test) predicted *het* genes exhibited coordinated upregulation at 3 h in N. crassa, during which nuclei from opposite mating types fused with the phase shift from dikaryotic to diploid, although the overall increase was not significant (*P = *0.053, one-sample *t* test versus 0). A second coordinated upregulation at stage 4 was observed for 16 predicted *het* genes, which is significant (*P = *0.0021, Fisher exact test) in contrast to only a third of the genome (3,425/9,717) that was upregulated, during meiotic sporulation, in N. crassa. Such coordination was not observed among predicted *het* genes in C. globosum.

10.1128/mBio.02119-19.3 FIG S3 Molecular phylogenies and sexual development expression profiles of heterokaryon incompatibility genes in N. crassa and C. globosum. (A) RAxML tree based on the best-scored alignment by SATé II identifies three major clades of *het* homologs between N. crassa and C. globosum, including orthologs of two N. crassa HET-C genes, members of the TOL-HET14 Bayesian phylogeny based on the best-supported SATé II alignment with focused sequences (B), and members of the HET-6/13/15 Bayesian phylogeny based on the best-supported SATé II alignment with focused sequences (C) (thick branches are supported with a posterior probability of ≥0.95). For Bayesian analyses, trees were sampled every 1,000th generation over four chains for 2,000,000 generations. One thousand trees obtained prior to convergence were discarded before computing a 50% majority-rule consensus of the remaining trees. (D) Expression profiles of *het* genes during sexual development in N. crassa (average expression, dashed black curve). (E) Expression profiles for *het* genes during sexual development in C. globosum (average expression, dashed black curve). (F) Expression of selected *het* genes in N. crassa and C. globosum during sexual development (relative to the lowest stage-specific expression). Bars indicate 95% confidence intervals. Download FIG S3, TIF file, 2.5 MB. Copyright © 2019 Wang et al. 2019 Wang et al. This content is distributed under the terms of the Creative Commons Attribution 4.0 International license.

### Bayesian networks for associations among conidiation, heterokaryon incompatibility, and sexual development.

**FIG 7:**
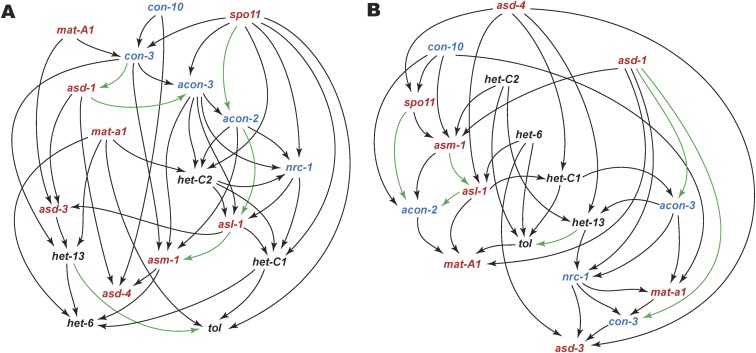
Bayesian networks during sexual development in Chaetomium globosum (A) and Neurospora crassa (B), relating associations among homologs of sexual development (red), conidiation (blue), and heterokaryon incompatibility (black). Edge connections represented in 50% or more of the likely models between the two networks (green), and edge directions are not necessarily the direction of regulation between the genes.

Our previous studies demonstrated that genes inferred to play central roles in the network model tend to be placed in the top-tier modules and have multiple edge connections (6, 64)—consistent with a conception that gene hubs in the network represent critical points (82, 83). Bayesian networks (BN) were constructed in this study to examine interactions of selected conidiation, *het*, and sexual development regulatory genes during perithecial development, including ascus development protein coding gene (*asd*) (Fig. 7 and Table S4). As expected, sexual development genes were positioned as core hubs of the process in BNs for both species. These core regulators interacted with both *con* and *het* genes. However, *con* genes were inferred as central in C. globosum (Fig. 7A), while *het* genes were inferred as central in N. crassa (Fig. 7B). Expression of *het* genes was highly coordinated during the pairing and fusion of two haploid nuclei from the opposite mating type in N. crassa. Consistent with that coordination, *het-C2* and *het-6* were attributed central roles and positioned as top-tier regulators of the N. crassa sexual development network, with dense direct associations with ascus and ascospore developmental genes. Associations among genes *acon-3*, *nrc-1*, and *con-3* and the two mating-type genes *mat A-1* and *mat a-1*—which have known roles in crossing and regulate *het* genes during early perithecial development in N. crassa (84)—were not inferred as central regulatory modules and were positioned as lower-tier elements in the network. The roles of *het* genes were inferred as less critical in the C. globosum network. The *het* genes—except for *het-C2—*were positioned low, with presumably less regulatory roles in the C. globosum network. Another contrast between the N. crassa and C. globosum networks is consistent with the life cycle divergence between them: mating loci *mat A-1* and *mat a-1* were placed as top-tier regulators in the C. globosum network, along with *asd-1* and *con-10*. This observation suggests a more centralized postcrossing role for mating-type loci in homothallic C. globosum than in heterothallic N. crassa, in which mating-type loci function to regulate the crossing process before the initiation of perithecial development.

10.1128/mBio.02119-19.8 TABLE S4 Input data matrix for the Bayesian network reconstruction, the model-averaged scores of edges and the structure matrix underlying the Bayesian networks depicted in Fig. 7. Download Table S4, XLSX file, 0.1 MB. Copyright © 2019 Wang et al. 2019 Wang et al. This content is distributed under the terms of the Creative Commons Attribution 4.0 International license.

### Expression of osmolarity responsive genes and secondary metabolism clusters.

**FIG 8:**
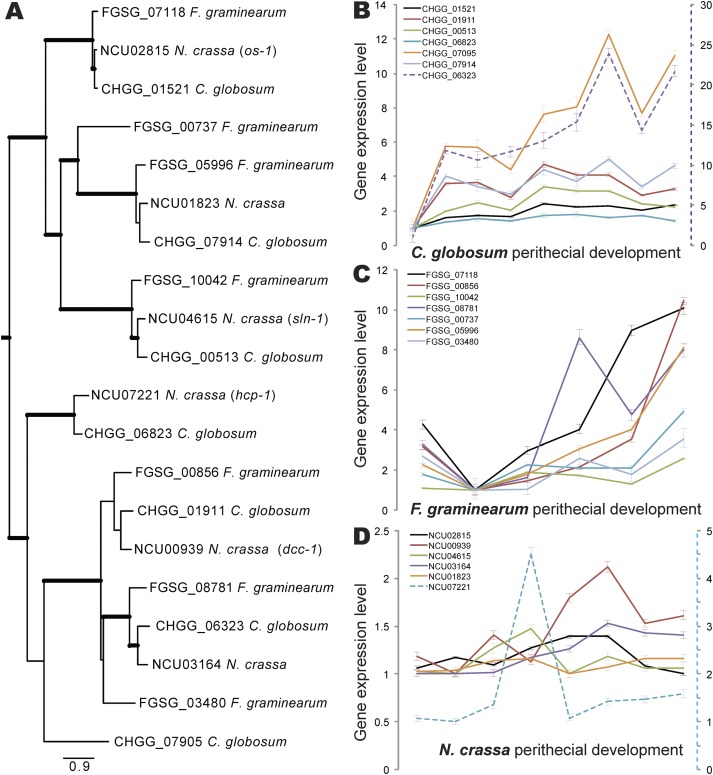
Phylogeny and comparative expression of hyperosmotic responsive histidine kinases (HKs) and related genes in Chaetomium globosum, Fusarium graminearum, and Neurospora crassa. (A) Phylogeny of the cellular HKs and related proteins. Trees were sampled every 1,000th generation over four chains for 2,000,000 generations. A total of 1,000 trees obtained prior to convergence were discarded before computing a 50% majority-rule consensus of the remaining trees. Thick branches received significant support (Bayesian posterior probability > 0.95). (B to D) Expression profiles of cellular HKs across sexual development, relative to the lowest stage-specific expression, in C. globosum (B), F. graminearum (C), and N. crassa (D). Bars indicate 95% confidence intervals. Note the second *y* axis (purple dashed line for C. globosum gene CHGG_06323; blue dashed line for N. crassa gene NCU07221), provided to quantify relative expression for one gene occurring on a different overall scale from other genes.

Phylogenetic analyses of the cellular hyperosmotic responsive histidine kinases (HKs) revealed seven C. globosum HKs that are homologous with six N. crassa HKs, including osmotic-1 (*os-1*), osmolarity two-component system protein (*sln-1*), development and carotenogenesis control-1 protein (*dcc-1*), and two-component system protein A (*hcp-1*) (Fig. 8A). During perithecial development, expression of these HKs was highly coordinately upregulated in C. globosum (Fig. 8B), as it is in F. graminearum (Fig. 8C), but was patternless in N. crassa (Fig. 8D).

A total of 41 secondary metabolic gene clusters (SMCs) were predicted in the genome of C. globosum CBS 148.51 by Department of Energy (DOE) Joint Genome Institute (JGI) and were further confirmed in this study using AntiSMASH (85) (Table S5). Among the 41 SMCs, 28 are composed of three or more genes, and 12 of these multigene SMCs exhibited highly synchronized expression across sexual development (Fig. S4A to L). Biosynthetic clusters for aureonitol (CHGG_00239 to CHGG_00246), chaetoglocin (10645 to 10649), and cochliodones (10019 to 10029) exhibited peak expression in mature perithecia—especially for cochliodones synthetic clusters, in which expression was upregulated only after the 4th stage of perithecial development, as asci and ascospores developed (Fig. S4G). Thirteen genes in C. globosum were reported by JGI as a single ortholog of SMCs in other genomes; nine of these exhibited upregulation during later perithecial development (Fig. S4M to O).

10.1128/mBio.02119-19.4 FIG S4 Expression profiles of genes within 15 secondary metabolism gene clusters across nine stages of sexual development, where expression has been quantified relative to the lowest stage-specific expression level for each gene. Download FIG S4, TIF file, 2.3 MB. Copyright © 2019 Wang et al. 2019 Wang et al. This content is distributed under the terms of the Creative Commons Attribution 4.0 International license.

10.1128/mBio.02119-19.9 TABLE S5 Forty-one secondary metabolism clusters predicted within the Chaetomium globosum genome in the JGI Mycocosm (119) database. Download Table S5, XLSX file, 0.1 MB. Copyright © 2019 Wang et al. 2019 Wang et al. This content is distributed under the terms of the Creative Commons Attribution 4.0 International license.

### Knockout phenotypes.

**FIG 9:**
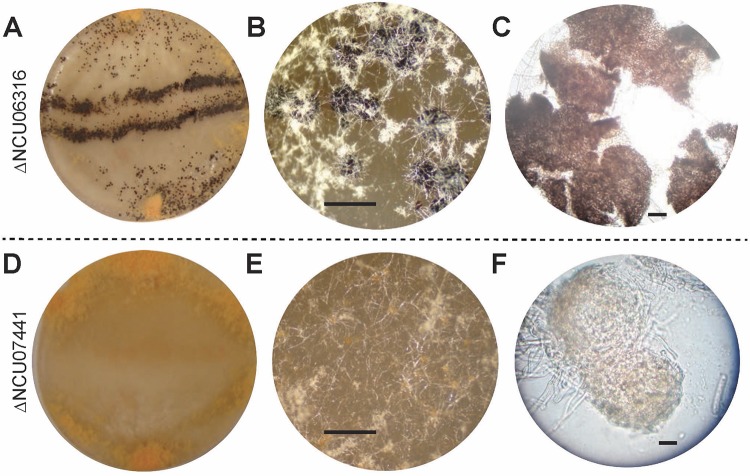
Knockout strains of unannotated genes exhibit mutant phenotypes on synthetic crossing medium, indicating critical functions in Neurospora crassa perithecal development. (A to C) The ΔNCU06316 knockout strain (FGSC20345 *mat a*; FGSC20346 *mat A*) forming bands of perithecia along the line of confluence after **A** and **a** strains were inoculated on the opposite sides of plates (A) and producing dark perithecia along these crossing bands (B) and squashed perithecium showing arresting perithecial development before production of asci and ascospores (C). The ΔNCU07441 knockout strain (FGSC15502 *mat a*; FGSC15503 *mat A*) not forming bands of perithecia along the line of confluence after **A** and **a** strains were inoculated on the opposite sides of plates (D) and producing abundant, light-colored protoperithecia on the plate surface (E) and squashed perithecium showing arresting protoperithecial development and exhibiting an abortive ascogenous center (F). Scale bars: 1 mm (B and E) and 50 μm (C and F).

In a comparison of C. globosum to N. crassa, 46 genes exhibited highly similar expression levels across sexual development, and 15 encode hypothetical proteins that have yet to be annotated with functions (Table S6). Knockouts of these unannotated genes from the public N. crassa knockout collection were phenotyped during sexual development; one (NCU06316) exhibited a knockout phenotype of early-stage arrested perithecial development (Fig. 9A to C). Homozygotes of NCU06316 knockouts (FGSC20345 *mat a* and FGSC20346 *mat A*) produced dark and enlarged perithecia but failed to produce asci and ascospores. An additional 12 late-responding light-induced genes and 4 early-responding light-induced genes (66) exhibiting differential expression regulation between the two species were also investigated. One gene (NCU07441; in the cross of **a** strain FGSC15502 and **A** strain FGSC15503) exhibited a knockout phenotype of arrest during protoperithecial development (Fig. 9D to F).

10.1128/mBio.02119-19.10 TABLE S6 Forty-six genes exhibiting highly similar gene expression patterns between Chaetomium globosum and Neurospora crassa across sexual development; 15 of the 46 are currently annotated as hypothetical proteins. Download Table S6, XLSX file, 0.1 MB. Copyright © 2019 Wang et al. 2019 Wang et al. This content is distributed under the terms of the Creative Commons Attribution 4.0 International license.

## DISCUSSION

Here we have profiled transcriptomics during sexual reproduction for Chaetomium globosum and compared it with closely related Neurospora crassa based on their shared similarity in sexual morphological development. We have revealed integrative activities among mating loci, environmentally responsive genes, and secondary metabolism pathways during C. globosum sexual development. Highly coordinated regulation of mating loci suggests that these genes contribute to the further regulation of postcrossing development in self-compatible C. globosum. Upregulated expression of the cellular hyperosmotic responsive histidine kinases suggests that sexual development of C. globosum has become highly adapted to humid environments. The active regulation of clusters of secondary metabolism genes across sexual development suggests that active fungal defense of fruiting bodies from predation is an important aspect of sexual development in C. globosum.

Although C. globosum and N. crassa are adapted to very different ecologies (highly humid, even aquatic substrates versus heat-killed postfire vegetation), the morphology and sexual development of C. globosum and the genetic model N. crassa are highly similar. Consistent with this observation, it is not surprising that regulation of expression in many gene markers associated with development shows similar patterns between C. globosum and N. crassa. For example, genes in the melanin biosynthesis pathways that are critical for ascospore pigment production (86) exhibited highly similar upregulation during ascospore development in *Chaetomium* and *Neurospora* species.

Efficient gene manipulation requires adequate disruption of high nonhomologous random recombination against foreign DNA in C. globosum (87), an issue that is already addressed with an efficient knockout approach in N. crassa (68). Taking advantage of the well-annotated N. crassa genome and N. crassa knockout mutants available from a systematic knockout program, we were able to assess possible functions of some of these genes in C. globosum. Two unannotated genes that exhibited similar degrees of expression regulation between C. globosum and N. crassa showed knockout phenotypes in sexual development in N. crassa, making them interesting regarding genetics of sexual development in C. globosum.

Although genetic background in mating type loci has been intensively studied for both self-incompatible and self-compatible lifestyles (42, 88–90), little is known about how such divergence in genetic settings play different roles in sexual development regulation between the two lifestyles. Mating-type loci behave in a highly coordinated fashion through the whole sexual reproduction process in self-compatible C. globosum, and high coordination was also observed for pheromone receptors and precursors (91). Sordaria macrospora—a species closely related to N. crassa and C. globosum—has provided great insights into the genetics of the self-compatible lifestyle. In S. macrospora, mating-type genes are required for the initiation of sexual reproduction (protoperithecia) and likely regulate fruiting body development and ascosporogenesis (92). In S. macrospora, mating-type genes are not required for protoperithecial development but are required for sexual reproduction and likely regulate ascosporogenesis and later steps in perithecium development (48, 63). In the heterothallic and pseudohomothallic species of *Neurospora*, neither significant upregulation of the *mat A* mating-type loci nor coordinated expression of the *mat A* and *mat a* loci has been observed during perithecial development. The divergence in expression activities of the mating-type loci between these self-compatible and self-incompatible species likely arose as a shift in the major roles of mating-type genes in self-compatible species: a loss of a role in the regulation of mating and crossing in self-incompatible species, perhaps accompanied by an enhanced role in the regulation of development of fruiting bodies in self-compatible species. In the reconstructed BN, the mating-type loci in C. globosum tightly associated with sexual developmental genes, whereas in the N. crassa BN, mating-type loci were loosely associated with *het* and *con* genes, suggesting a loss of regulatory roles during ascus and ascospore development. Experimental research perturbing the mating-type loci and mitogen-activated protein kinase (MAPK) signaling pathways in postcrossing stages of perithecial and ascospore development from both species would further clarify the genetic divergence in mating-type gene regulatory action and the concomitant changes in fungal life history.

There are many commonalities among filamentous fungi in how they respond to environmental stresses by production of resistant sexual reproductive structures and ascospores (70). Genes responsive to light, oxygen, and humidity exhibited dissimilar expression patterns among C. globosum, F. graminearum, and N. crassa during sexual reproduction, indicating that potentially common processes of perithecial development are responsive to different environmental conditions among these fungi. While we did not assay expression under differing environmental conditions in this study, we did maintain a homogeneous environment in which species with differing genetics were cultured; interestingly, genes that regulate fungal response to environmental factors such as reactive oxygen species (ROS) were expressed in a species-specific manner. Cellular hyperosmotic responsive histidine kinases (HKs) are recognized as transducers of diverse environmental signals (93, 94), and our evolutionary analyses revealed additional copies of these HKs in C. globosum that are homologous to those in N. crassa and F. graminearum. Coordinately upregulated expression of all HKs in C. globosum and F. graminearum is consistent with observation of preferences for high humidity and the demonstrated role of water in spore release in these fungi (20, 95). We observed low fold changes that composed mixed expression patterns for these HKs, including *os-1*, *hcp-1*, *sln-1*, and *dcc-1*, during sexual development in N. crassa, consistent with a conception that humidity is not critical for the propagation of the postfire, air-dispersed fungus N. crassa.

Nevertheless, humidity affects conidiation in N. crassa (96), and expression of HKs does respond coordinately to environmental humidity in some stages of the life history. Two of the HKs—*hcp-1* (75) and *dcc-1* (97)—are conidiation related. The degrees of expression of these HKs were divergent during N. crassa conidium germination between different media (64). All these HKs exhibited similar patterns of downregulation when N. crassa was cultured on Bird medium that was specifically formulated to induce conidiation. In contrast, these HKs—except *hcp-1*—were generally upregulated for cultures on a natural, carbon-rich, nitrogen-poor maple sap medium that supports both asexual and sexual reproduction. These findings indicate that these genes may be key components of the network that regulates the environmentally mediated asexual-sexual switch in fungi.

Another exciting aspect of sexual development in C. globosum is the activities of its numerous pathways devoted to synthesis of secondary metabolites. Some secondary metabolites are part of the fungal protection and defense system (98–100). Thus, it is not surprising that many SMCs were upregulated in response to the disturbance step implemented to synchronize perithecial development. Eleven SMCs exhibit highly coordinated expression dynamics across sexual development. Ten of these SMCs are expressed at a high level before the fungus is equipped with physical defenses such as thick-walled cells and septae. Exceptions include gene clusters that encode enzymes for the synthesis of cochliodones. Expression of these genes increases markedly only after 48 h of postcross perithecial development—concomitant with the development of asci and ascospores. Our observation of coordinated upregualtion of mating loci, melanin synthesis pathways, and many secondary metabolism pathways during C. globosum sexual development is consistent with recent studies reporting highly associated activities among mating loci and secondary metabolism pathways during sexual development of pathogenic fungi (101–103). Our discovery of stage-specific expression in the majority of SMCs in C. globosum will be useful to the development of strategies to manipulate the growth and development of these fungi for high production of secondary metabolites.

Lack of asexual reproduction in C. globosum remains a mystery—especially when it is noted that some members of the genus *Chaetomium*—including C. globosum species complex—do produce abundant conidia (16). Our finding that orthologs of major conidiation genes exist and are actively expressed during sexual development in C. globosum calls for further investigation of the ecology, evolution, and genetics of fungal conidiation and other sporulation pathways. In natural settings, fungi quickly occupy a substrate by inducing rapid growth and producing myriad fragile, genetically uniform asexual spores (conidia). In contrast, sexual reproduction is usually a slow process, during which fruiting bodies facilitate the production and dissemination of hardy propagules that have diversified genetics due to recombination (104). However, conidiation has never been observed for many fungi, including the homothallic fungal model Sordaria macrospora (105).

Three hypotheses, including yet-to-be-discovered asexual reproduction, loss of key conidiation genes or other mutation leading to loss of conidiation, and unfavorable combination of heterokaryon incompatibility genes, have previously been suggested to explain the lack of conidiation in S. macrospora (105). Like the S. macrospora genome, the C. globosum genome hosts homologs of most conidiation-related genes as annotated in N. crassa, but orthologs of conidiation genes *con-6*, *con-8*, and *con-13* were not found in the C. globosum genome. Mutants of *con-8* and *con-13* exhibited defective growth of aerial hyphae and conidium production but normal vegetative growth in N. crassa (106–108). In N. crassa, expression of *con* genes has long been known in all three sporulation pathways, including the production of macroconidia, microconidia, and ascospores, suggesting that their roles are typically not strictly relegated to any single sporulation process and that they are independently activated during each sporulation (109).

Investigation of the behavior of *het* genes during conidiation for these fungi that produce no conidia is challenging. Incompatibility and resulted programmed cell death are induced by nonallelic interactions between two separate *het* loci (54), and mild incompatibility can lead to a loss of ability to conidiate (105, 110, 111). In N. crassa, the nonallelic interactions between *het-C* and *pin-C* are critical for nonself recognition and programmed cell death (112). However, we found no ortholog of Pin-C in C. globosum, suggesting the loss of one means of incompatibility in C. globosum due to the possible absence of interactions between Het-C and the missing Pin-C.

Similar to the case in Sordaria macrospora, C. globosum homologs of N. crassa TOL and HET-6 exhibit a large amino acid sequence dissimilarity to N. crassa TOL and HET-6. C. globosum homologs of these genes may have lost their functions in regulating heterokaryon incompatibility, and the loss of conidiation probably became a mild cost as being “cryptic heterokaryon incompatibility” in order to maintain both mating types in the same hyphae, as speculated for S. macrospora (105). In fact, *mat* genes are also heterokaryon incompatibility genes in N. crassa (113, 114). However, interactions among conidiation genes and heterokaryon incompatibility during sexual spore production could provide some clues regarding possible conflicts between the two functional groups. Reconstructed Bayesian networks relating the associations of expression among sexual development, conidiation, and heterokaryon incompatibility genes suggest that conidiation and heterokaryon incompatibility relate differently to sexual development in N. crassa and C. globosum. Namely, heterokaryon incompatibility was tightly associated with regulation of sexual development of asci and ascospores in N. crassa, whereas it was not involved in the regulation of sexual development in C. globosum, indicated by a lack of direct connections with sexual regulators. However, the preservation and frequent upregulation of these *het* genes during ascospore maturation suggest that these genes operate in unknown but important roles in sexual development in both N. crassa and C. globosum.

Interestingly, expression of conidiation genes is highly associated with the expression of sexual regulators during asci and ascospore development in C. globosum, and some of these associations—including *con-3* and *asd-1*, *spo11* and *acon-2*, *asd-1* and *acon-3*, and *acon-2* and *asl-1*—are conserved between the N. crassa and C. globosum BNs. Apparently, as the production of ascospores commences, these conidiation genes are regulated by divergent developmental mechanisms between C. globosum and N. crassa. In C. globosum, conidiation-associated genes and heterokaryon incompatibility loci might have lost their coregulation along with losing heterothallism. There has been speculation of a shared genetic basis among different sporulation pathways (109). Nevertheless, the roles of conidiation genes in ascospore production are barely characterized in N. crassa. Because conidia are essential to starting the fertilization process, it is challenging to study the role of *con* genes during sexual development in heterothallic N. crassa. Our observation would promote C. globosum as an alternative model to study shared regulatory mechanism among different sporulation pathways and conidiation genes’ roles in ascospore production in these fungi. Additional genetic data for these genes and how they interact with heterokaryon incompatibility gene during asexual growth in self-compatible and non-conidium-producing C. globosum and S. macrospora, self-compatible and conidium-producing F. graminearum, and self-incompatible and conidium-producing N. crassa would be highly informative. Comparative genomics between conidiation and nonconidiation species in the species complex of C. globosum could also illuminate why some of these fungi have lost their proclivity or their ability to reproduce asexually.

### Conclusion.

Comparative gene expression of the sexual development between C. globosum and closely related N. crassa revealed that mating-type loci are expressed highly coordinately in C. globosum and appear to play roles in regulating postcrossing sexual development divergent from their roles as regulators of mating in N. crassa. This study calls for further investigation of the means by which conidiation genes have evolved to interact with heterokaryon incompatibility genes in diverse fungal models to understand why conidiation has not yet been observed for some of the fungi. We have shown that environmental responses to humidity and secondary metabolite synthesis pathways are actively regulated during C. globosum sexual reproduction. Some pathways are highly expressed during sexual reproduction producing resistant perithecia and ascospores in C. globosum, providing useful information for diagnostic and treatment purposes regarding this pathogenic fungus.

## MATERIALS AND METHODS

### Induction of synchronous perithecial development.

The genome-sequenced strain of Chaetomium globosum (CBS 148.51) was cultured on carrot agar (CA [115]), enabling comparison to related studies conducted on *Neurospora* and *Fusarium* species (5, 6, 56, 115). In contrast with species of *Neurospora* and *Fusarium*, C. globosum produces no conidia. Culture of C. globosum on a medium with a low concentration of simple sugars—such as CA medium—represses germination of C. globosum ascospores, which otherwise can be used to induce a large amount of synchronic growth. To culture enough tissue exhibiting synchronized perithecial development, hyphae of C. globosum were inoculated in 200 ml of liquid CA in 500-ml flasks. The flask cultures were incubated at 27°C on a 100-rpm shaker under constant light. Ten-day liquid cultures were filtered with a sterilized single-layer miracloth (Calbiochem), and abundant hyphal elements were harvested from the filtrate (Fig. S1). Two milliliters of the filtrate was plated out on a cellophane membrane covering solid CA in a petri dish (9 cm in diameter) and then incubated at 27°C under constant white light in a refrigerated incubator (VWR Signature diurnal growth chamber). Plates with apparent protoperithecia were gently disturbed with a glass microbiological spreader to mimic crossing protocols applied to other heterothallic systems in previous studies; such a disturbance is known to be critical for setting a synchronous start time for perithecial development in F. graminearum (7). Fungal tissues were collected by removal of cellophane membranes at the protoperithecial stage (0 h, right before disturbance), as well as at 2, 24, 48, 72, 96, 120, 144, and 168 h after disturbance (stages 2 to 9). Tissue samples were flash frozen in liquid nitrogen and stored at −73°C. Tissues collected from a single plate were used as one biological replicate. At least three biological replicates were prepared for every sampled time point.

### RNA isolation and transcriptome profiling.

Total RNA was extracted from homogenized tissue with TRI reagent (Molecular Research Center) as described in reference 116. mRNA was purified using Dynabeads oligo(dT) magnetic separation (Invitrogen). Preparation of cDNA for sequencing followed the Illumina mRNA sequencing sample preparation guide. The quality of cDNA samples was verified with a bioanalyzer (Agilent Technologies); 22 quality samples—including 5 purely technical replicates—were sequenced at the Yale Center for Genomics Analysis (YCGA).

### Data acquisition and analysis.

Seventeen sequencing libraries were produced from purified total RNA samples, 76-bp single-end sequenced on an Illumina HiSeq 2500 using the TruSeq stranded protocol, generating an average of 22 million single-end reads per library (Table S2). Reads were aligned using Tophat v.2.1.1 (117) using the very-sensitive preset. Only the reads that mapped to a single unique location within the genome, with a maximum of two mismatches in the anchor region of the spliced alignment, were reported. We tallied aligned reads with the program HTSeq v0.6.1p1. An additional five technical replicates were sequenced to ensure quality control among serial sequencing runs. Statistical analysis of gene expression levels based on the tallies of reads for each gene was conducted with LOX v1.6 (118). Raw reads that mapped ambiguously or to multiple loci were excluded from LOX input. Previously generated data on gene expression during sexual development in N. crassa (GSE41484 [6]) and in F. graminearum (GSE61865 [56]) were compared to the new C. globosum gene expression data.

### Gene orthology assessment and ancestral gene expression estimation.

To identify orthologs, protein and nucleotide sequences were downloaded from the JGI genome database (119). Predicted protein sequences were used to identify single-copy orthologs (cluster) with ReMark (120), specifying the BLOSUM62 amino acid transition matrix and an inflation factor of 1.6. The ortholog set was compared to those reported in the 8th InParanoid database (121) for missing or misidentified clusters; any contrasting results were further verified by manually conducting phylogenetic analyses on sequences obtained from exhaustive reciprocal BLAST searches. For phylogenetic analyses, amino acid sequences were aligned using SATé-II (122) specifying MAFFT as the aligner, MUSCLE as the merger, and RaxML as the tree estimator under the WAG model. The alignment with the best score was retained for analysis with MrBayes 3.2 (123) by Metropolis-coupled Markov chain Monte Carlo with a mixed model for the amino acid molecular evolution. Clades with a posterior probability (PP) of >0.95 were deemed significantly supported. Gene expression patterns that were ancestrally retained, convergent, or divergent were classified with ancestral expression reconstructed as described in reference 7. Briefly, the fold change between stages and the molecular evolutionary tree of selected species were supplied as input files to the Continuous Ancestral Character Estimation (CACE [124, 125]) tool in the Discovery Environment Application list in CyVerse (126–129), which provided ancestral changes in expression across adjacent stages at all internal nodes for every ortholog set.

### Bayesian network reconstruction.

Bayesian networks (130) based on time series expression data express significant coregulatory posterior probability with each edge and enumerate associations of a gene with other genes by the degree at each vertex. The direction of the edge in a BN network is not necessarily the regulatory direction, especially without incorporation of specific genetic perturbation data. However, evidence of the centrality of gene function is strengthened with dense direct network connections, presumably because multiple genes are coregulated for a specific developmental purpose. Biological networks were modeled using the Bayesian Network web server (130) supplied with perithecial development expression data for N. crassa and C. globosum separately. To scale changes between serial sample points appropriately for Bayesian Network inference, they were quantified as

$$\left\{ \begin{array}{c} \left(\left(x_{t+1}-x_{t}\right)/\min\left[x_{t},x_{t+1}\right]\right)/2,\;\;\;x_{t+1}-x_{t}<2\\ \log_{2}\left(\left(x_{t+1}-x_{t}\right)/\min\left[x_{t},x_{t+1}\right]\right),\;x_{t+1}-x_{t}\geq 2 \end{array}\right.$$

where *x_t_* is the relative expression level as quantified by LOX (118) at stage *t* ∈1.8 for N. crassa, and *t* ∈1.9 for C. globosum. The relative expression levels of two stages were compared for the minimum (min). Global structure learning settings were retained at default settings. Each network depicted is the 50% majority consensus of the 100 highest-scoring models that retain edges exceeding a selection threshold of 0.5, performed without imposing any structural constraints.

### Functional enrichment and secondary metabolic gene clusters.

Functional annotation of statistically significantly differentially expressed genes in metabolic pathways was gathered via the biochemical pathway and annotation data from the Kyoto Encyclopedia of Genes and Genomes (KEGG [131]). Gene ontology (GO) enrichment analysis was performed with Panther provided by Gene Ontology Resource (132). Functional annotation was further checked for genes of interest using the FungiDB database (133). Forty-one SMCs indicated within the C. globosum genome by entries in the JGI database were further confirmed using AntiSMASH (85). Genome-wide expression patterns were clustered with a hierarchical algorithm of Euclidean distance using Morpheus (https://software.broadinstitute.org/morpheus).

### Nucleic acid manipulation and genetic transformation.

Knockout strains were constructed for more than 9,600 genes via the Neurospora crassa knockout project (68, 134), obtained from the Fungal Genetics Stock Center (FGSC [135]). Deletion of target genes in the knockout mutants was then verified by PCR in our lab using the methods previously described (6, 136, 137). Each knockout strain that exhibited a significant morphological phenotype was crossed with the wild-type strain. Cosegregation of the observed phenotype with deletion of the gene in the offspring was verified to ensure that the intended deletion was responsible for the mutant phenotype (6, 136, 137).

### Accession number(s).

All gene expression data were deposited in the Gene Expression Omnibus database (accession number GSE131190).
