# Supplementary figures and images for: Integrative Activity of Mating Loci, Environmentally Responsive Genes, and Secondary Metabolism Pathways during Sexual Development of Chaetomium globosum
Source: mBio. 2019 Dec 10;10(6):e02119-19. doi: 10.1128/mBio.02119-19 (PMC6904875; doi:10.1128/mBio.02119-19)

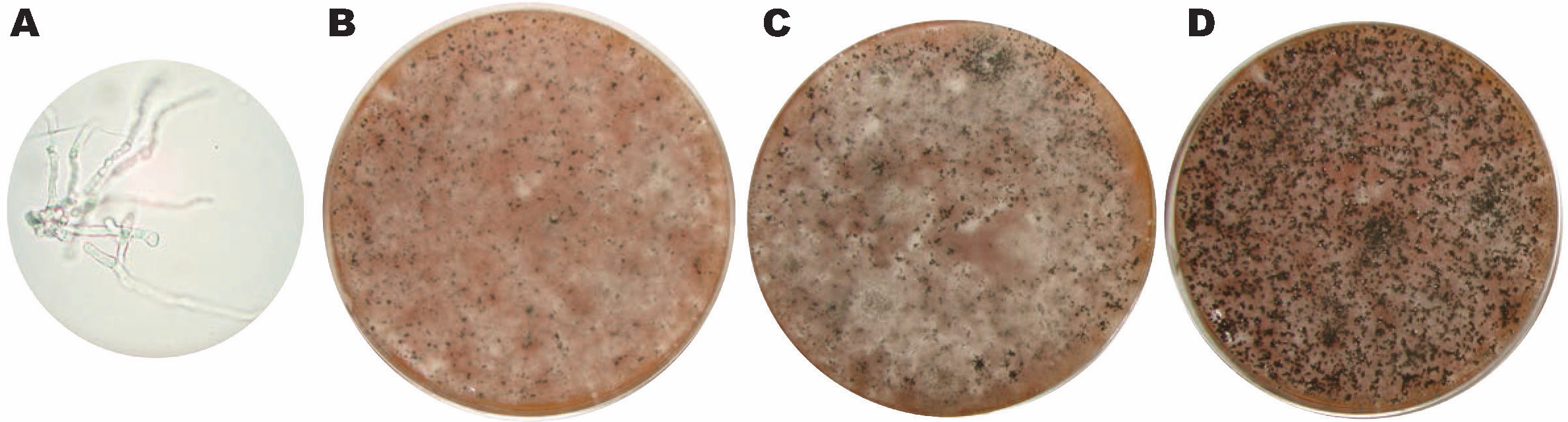

Supplement: FIG S1 [file mBio.02119-19-sf001.tif]

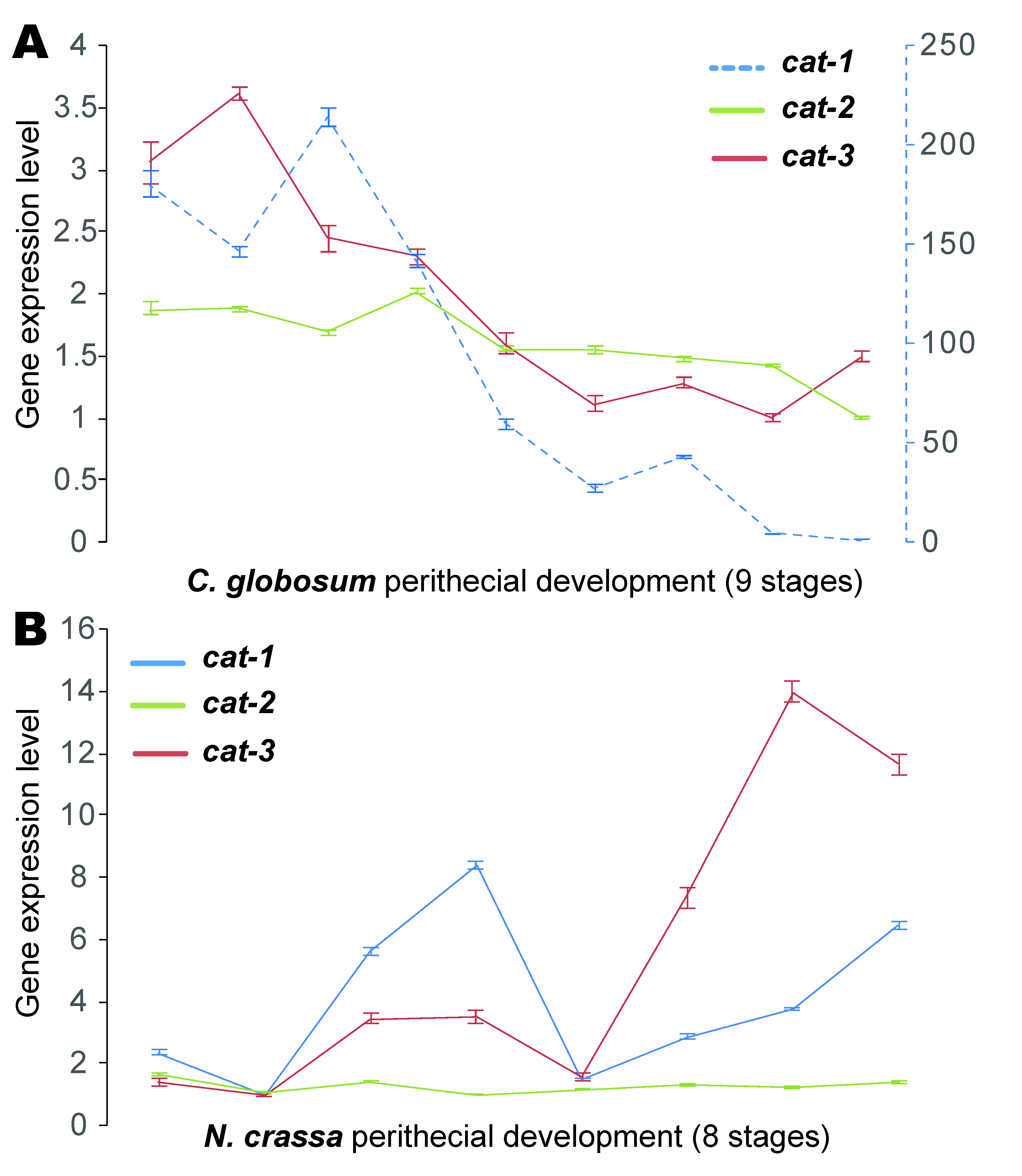

Supplement: FIG S2 [file mBio.02119-19-sf002.tif]

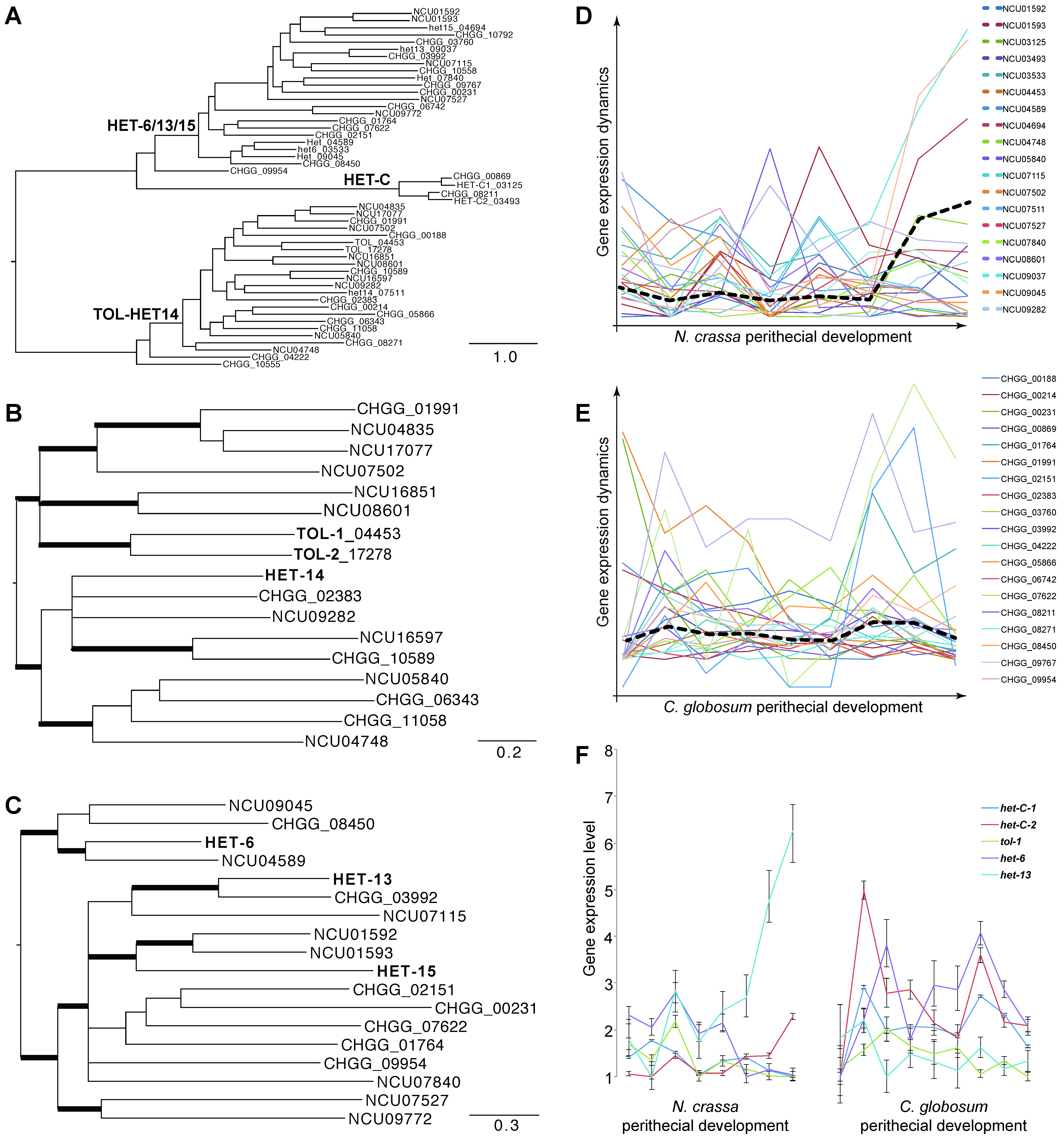

Supplement: FIG S3 [file mBio.02119-19-sf003.tif]

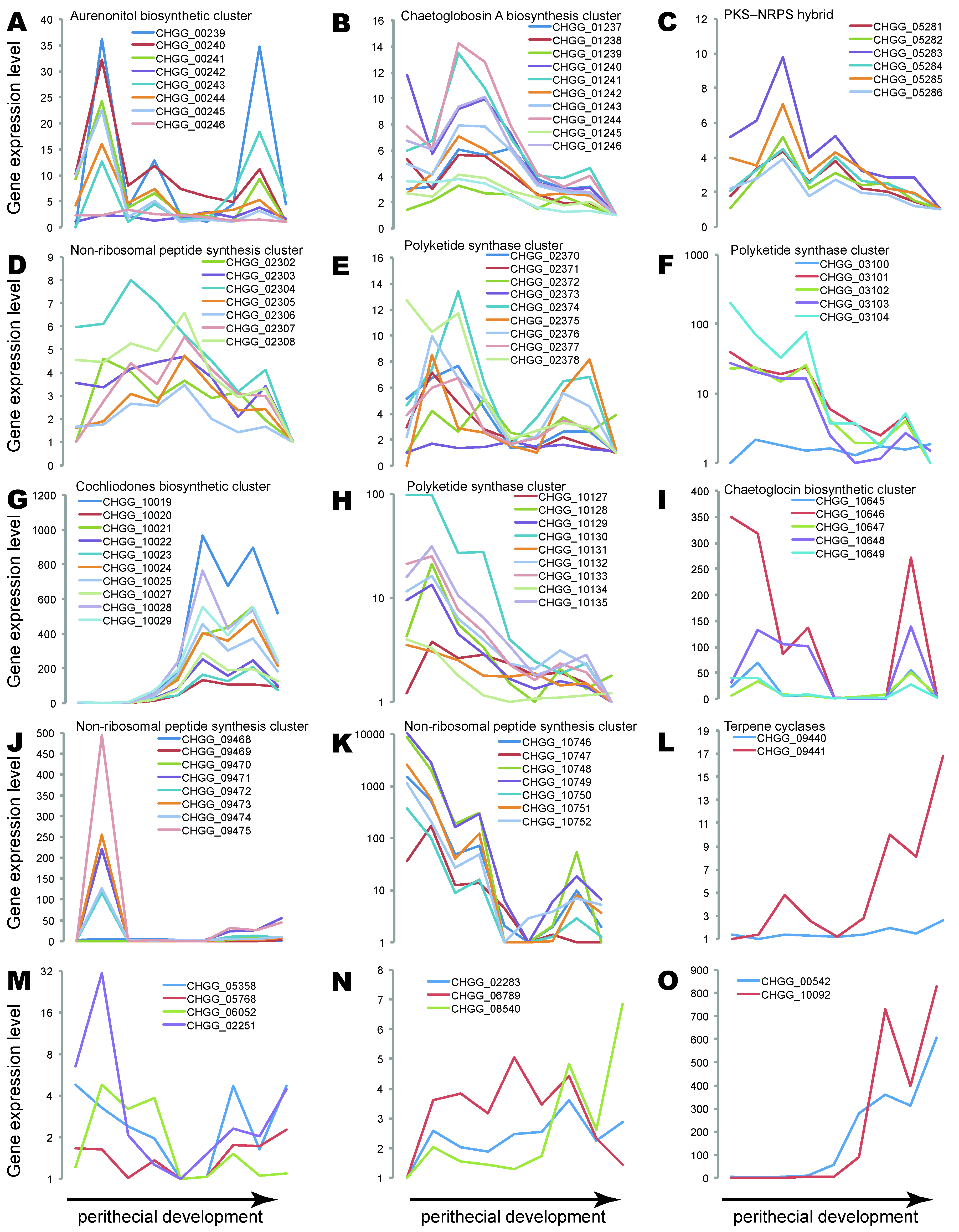

Supplement: FIG S4 [file mBio.02119-19-sf004.tif]
